# Usefulness of PET With [^18^F]LBT-999 for the Evaluation of Presynaptic Dopaminergic Neuronal Loss in a Clinical Environment

**DOI:** 10.3389/fneur.2020.00754

**Published:** 2020-08-21

**Authors:** Maria-Joao Ribeiro, Johnny Vercouillie, Nicolas Arlicot, Clovis Tauber, Valérie Gissot, Karl Mondon, Laurent Barantin, Jean-Philippe Cottier, Serge Maia, Jean-Bernard Deloye, Patrick Emond, Denis Guilloteau

**Affiliations:** ^1^UMR 1253, iBrain, Université de Tours, Tours, France; ^2^CHRU, Tours, France; ^3^Inserm CIC 1415, CHRU, Tours, France; ^4^Zionexa, Saint-Beauzire, France

**Keywords:** Parkinson's disease, dopamine, DAT, PET, radiopharmaceutical

## Abstract

**Purpose:** The density of the neuronal dopamine transporter (DAT) is directly correlated with the presynaptic dopaminergic system injury. In a first study, we evaluated the brain distribution and kinetics of [^18^F]LBT-999, a DAT PET radioligand, in a group of eight healthy subjects. Taking into account the results obtained in healthy volunteers, we wanted to evaluate whether the loss of presynaptic striatal dopaminergic fibers could be estimated, under routine clinical conditions, using [^18^F]LBT-999 and a short PET acquisition.

**Materials and methods:** Six patients with Parkinson's disease (PD) were compared with eight controls. Eighty-nine minutes of dynamic PET following an intravenous injection of [^18^F]LBT-999 were acquired. Using regions of interest for striatal nuclei, substantia nigra (SN), cerebellum, and occipital cortex, defined over each T1 3D MRI, time–activity curves (TACs) were obtained. From TACs, binding potential (BP_ND_) using the simplified reference tissue model and distribution volume ratios (DVRs) using Logan graphical analysis were calculated. Ratios obtained for a 10-min image, acquired between 30 and 40 min post-injection, were also calculated. Cerebellum activity was used as non-specific reference region.

**Results:** In PD patients and as expected, striatal uptake was lower than in controls which is confirmed by BP_ND_, DVR, and ratios calculated for both striatal nuclei and SN, significantly inferior in PD patients compared with controls (*p* < 0.001).

**Conclusions:** PET with [^18^F]LBT-999 could be an alternative to assess dopaminergic presynaptic injury in a clinical environment using a single 10 min acquisition.

## Introduction

The core symptoms of parkinsonian syndrome (akinetic rigid syndrome, tremor syndrome, etc.) may not always be present in a single patient (1). Although Parkinson-like symptoms are often observed in many diseases, the positive diagnosis of Parkinson's disease (PD), despite consensus disease criteria, remains inaccurate. For example, the accuracy of clinical diagnosis of PD varies between 74% (diagnostic performed by non-experts) and 84% (diagnosis performed by movement disorders experts), and it has not significantly improved in the last 25 years (2).

It has been known for many years that the dopaminergic neurotransmitter system plays a major role in movement disorders, and particularly in PD, as well as in dementia with Lewy body (DLB) (3–7).

The dopamine neuronal transporter (DAT) is responsible for the re-uptake of dopamine from the synaptic cleft to the pre-synaptic neuron and could be used to evaluate the integrity of pre-synaptic dopaminergic fibers (8–13). In this field, molecular imaging exploration of the DAT is a marker of pre-synaptic neuronal integrity and could be very useful for the early diagnosis and follow-up of PD (14) and DLB (6). Currently, in our nuclear medicine department, as in many others, the evaluation of the pre-synaptic dopaminergic neuron's degeneration is explored in clinical routine with a SPECT DAT ligand, the FP-CIT labeled with iodine-123 (15, 16).

However, using a tracer labeled with iodine-123, the protection of the thyroid gland is required beforehand. This protection can be performed with potassium perchlorate 20 min before injection or potassium iodine 1 h before [^123^I] FP-CIT injection. Then, between the administration of the radiotracer and the SPECT acquisition, a minimum period of 3 h is necessary. Finally, the typical total scan time is around 30 and 45 min depending on the imaging gamma camera used (16).

Thus, the ability to evaluate the integrity of the pre-synaptic dopaminergic system with a radiotracer that does not require a protection of the thyroid—with a relatively short delay between injection and imaging procedure, as well as a fast acquisition—becomes a priority.

For PET imaging, several DAT radioligands labeled with carbon-11 have been proposed for many years. However, since [^18^F] can be more largely used for clinical purposes than [^11^C] owing to its longer physical period (110 vs. 20 min), there is an increasing need to produce high-performance radioligands labeled with fluorine-18.

Cocaine derivatives have high affinity for DAT and, among these ligands, the [^18^F] (2S,3S)-methyl-8-((E)-4-fluorobut-2-en-1-yl)-3-(p-tolyl)-8-azabicyclo[3.2.1]octane-2-carboxylate, or [^18^F]LBT-999, has been developed for DAT brain imaging. *In vitro* studies have demonstrated that LBT-999 has high affinity for DAT (*K*_*d*_ = 9 nM, *B*_max_ = 17 pmol/mg protein) and very high selectivity for DAT vs. serotonin and noradrenaline transporters (17, 18).

*In vivo* kinetic PET studies using this radioligand in baboons have showed a fast and very high uptake of the tracer in the striatum, with a plateau at 30 min post-injection and a maximal putamen/cerebellum ratio of 30 (19).

In a previous study, our group demonstrated that the cerebral distribution of [^18^F]LBT-999 on healthy controls was consistent with DAT density and in good agreement with other PET DAT radioligands in the human brain (20).

The use of a PET semi-quantitative index is very interesting to compare populations or to analyze the evolution of the disease or the effectiveness of a treatment. The validated methods for DAT quantification based on a simplified reference tissue model (SRTM) or a Logan analysis rely on PET a dynamic acquisition, which consistently limits their use in clinical settings where dynamic acquisitions are not always feasible. Thus, we not only evaluated whether [^18^F]LBT-999 would be useful in estimating presynaptic dopaminergic injury but also if this could be done using a 10-min PET acquisition.

## Materials and Methods

### Radiosynthesis

No-carrier-added [^18^F]LBT-999 was prepared according to Dollé et al. (17) and Sérrière et al. (21). [^18^F]LBT-999 was produced with a 35 ± 8% radiochemical yield, radiochemical purity higher than 98%, and a mean molar activity of 143 ± 123 GBq/μmol.

### Subjects

PET studies with [^18^F]LBT-999 were performed in a group of six PD patients (age: 69 ± 7 years, Hoehn and Yahr: stages I–II without atypical signs) and compared with our group of eight healthy controls (HC) (age: 69 ± 9 years) initially studied with this same radioligand (20).

In order to meet French regulations and laws on biomedical research, the study was approved by the regional Ethic Committee and the French Agency for Safety and Security for Medical Devices. All the subjects were affiliated to the French social security system and gave their written informed consent after the nature of the procedure had been fully explained.

### PET and MRI Acquisitions

An 89 min list mode PET acquisition was acquired followed an intravenous injection of [^18^F]LBT-999 (3.6 MBq/kg; mean specific activity of 63.4 GBq/μmol at the moment of injection) using an Ingenuity Philips tomograph (Ingenuity TF64; Philips Medical Systems). The acquisition started just before the bolus injection to get the vascular peak. Acquired data were processed with the standard package delivered with the system (PET view software; Philips Medical Systems). For cerebral studies, a low-dose CT helical scan was performed at 80 keV and 40 mAs, delivering an irradiation dose of about 40 mGy × cm. PET sinograms were corrected for tissue attenuation, decay, scatter, and random radiation, and then they were reconstructed using a 3D iterative RAMLA algorithm in voxels of 2 × 2 × 2 mm^3^.

Brain MRIs were obtained for all subjects using a 3-T imager (Siemens Verio). T2-weighted images from each subject were used to reveal eventually brain lesions and signal abnormalities in the basal ganglia. In addition, a T1-weighted SPGR acquisition with inversion-recovery was performed to allow a 3D reconstruction of MRI images.

### PET Analysis

The time frames collected between 0 and 59 min after injection were summed to create an integrated image, which is used to perform the PET-MRI co-registration. To obtain time–activity curves (TACs) from 0 to 89 min post-injection, volumes of interest for caudate (Cd) and putamen (Pu) nuclei, substantia nigra (SN), occipital cortex, and cerebellum were defined over each T1 3D MRI, and after the PET-MRI co-registration displaced over PET images, using Pmod (version 3.4) software.

Using TACs (0–89 min) and Pmod software, binding potential (BP_ND_) using the SRTM and distribution volume ratio (DVR) obtained from Logan graphical analysis were calculated for striatal nuclei and SN. Ratios obtained for a 10-min image (acquired between 30 and 40 min post-injection) were obtained for the same regions. For all the calculations, the cerebellum activity was used as non-specific reference region.

### Statistical Analysis

All data are presented as mean ± SD. Mann–Whitney non-parametric *U*-test was used for comparison of tracers binding between PD patients and healthy controls. Statistics were performed using the XLStat software. All tests were two sided, with a significance set at *p* < 0.05.

## Results

**Figure 1:**
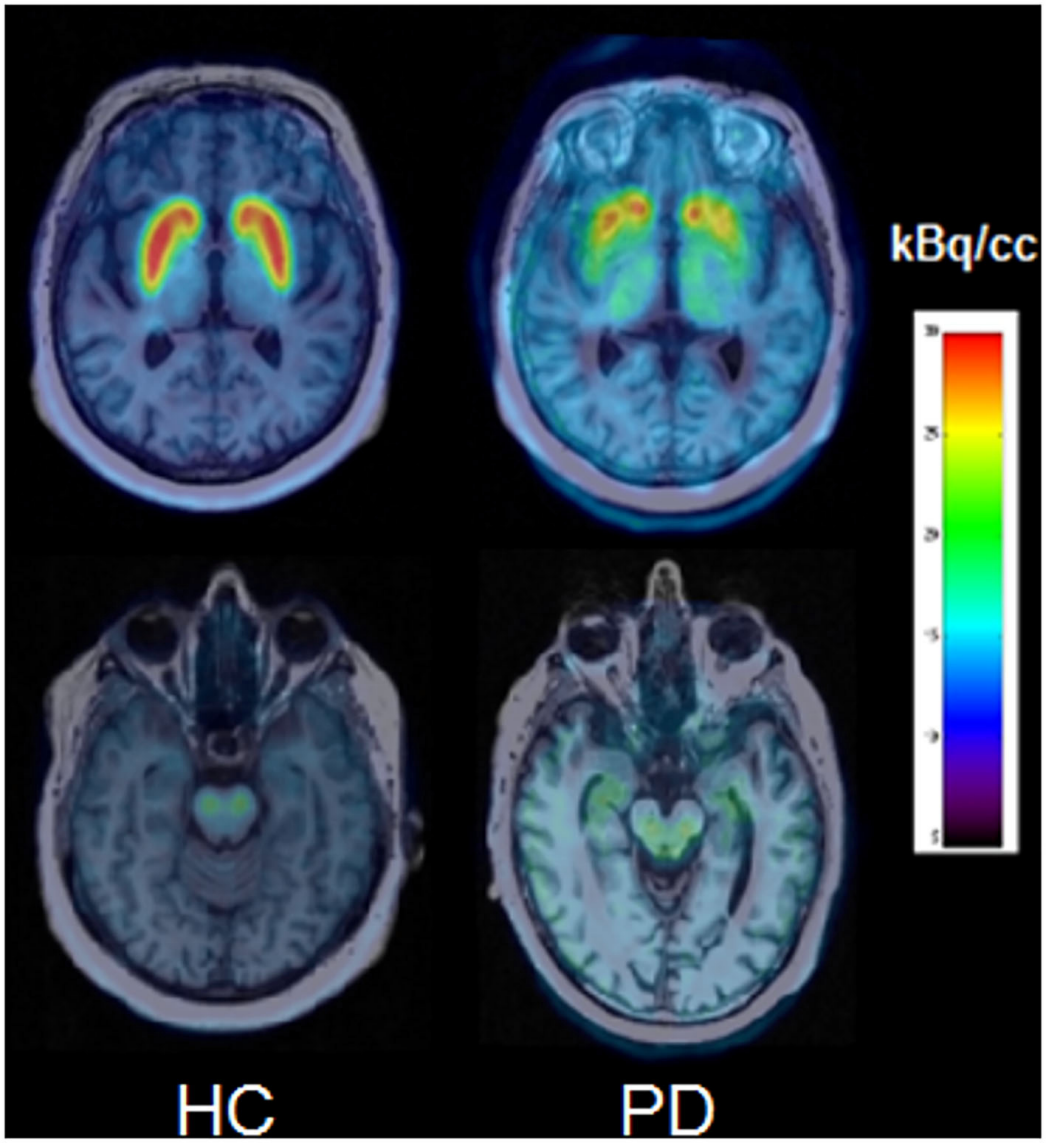
PET/MRI axial slices obtained between 30 and 40 min post-injection for a healthy control (HC) and a PD patient at the level of striatal nuclei **(top)** and midbrain **(bottom)**.

Figure 1 shows examples of axial PET/MRI fusion images obtained between 30 and 40 min post-injection in a healthy control (HC) and in a parkinsonian patient. A quite homogeneous and symmetrical striatal uptake for healthy controls was observed. In PD patients, the uptake is clearly reduced, especially on the putamen, and as expected, asymmetrical according to the clinically more affected side.

**Figure 2:**
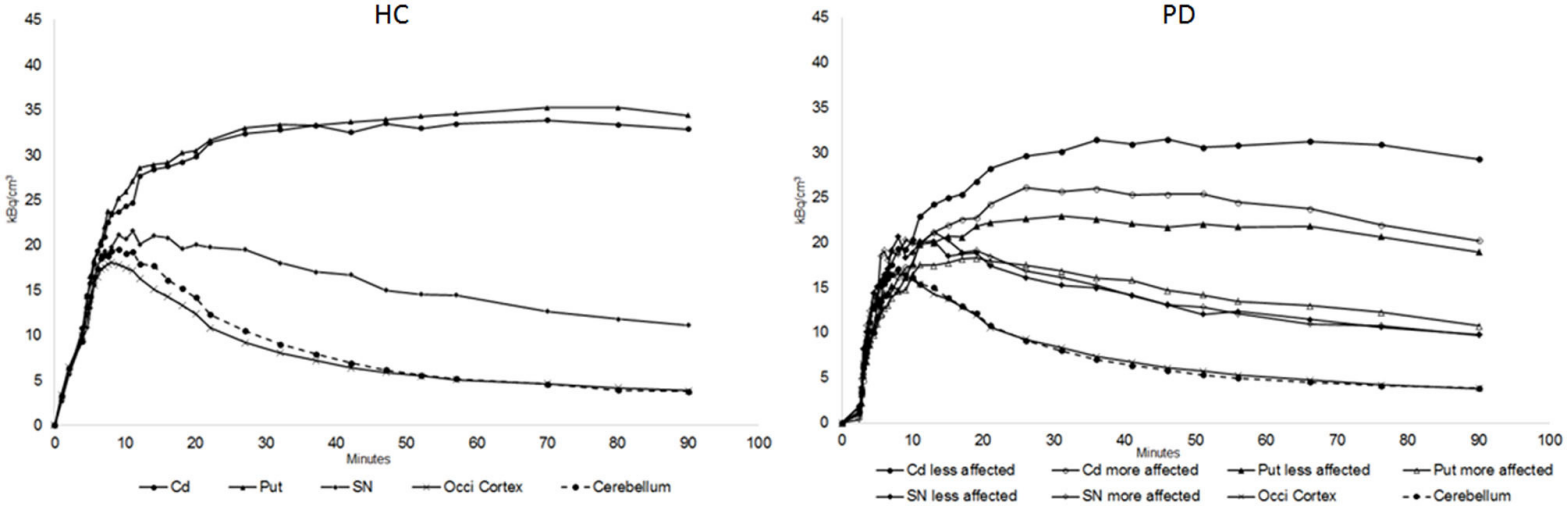
Average TAC of [^18^F]LBT-999 for caudate (Cd) and putamen (Put) nuclei, substantia nigra (SN), occipital cortex, and cerebellum for a healthy control (HC). For PD patients, TACs are presented for the more and less affected side for the same specific regions.

Figure 2 shows examples of TACs obtained for a HC and a PD patient. For the control, we present mean TACs corresponding to caudate nucleus, putamen, and SN. For the patient, the TACs are presented taking into account the more and less affected side of each structure. In the patient, the maximum striatal uptake is lower than that observed in the control. This is especially marked for the more affected putamen that shows a slow radioactive concentration decrease from 20 min post-injection until the end of the acquisition. For the less affected putamen and the more affected caudate nucleus after a plateau between 20 and 60 min post-injection, the radioactive concentration decreases progressively. The less affected caudate presents an uptake distribution quite similar to that of the striatal structures of the controls. For SN, the evolution of radioactivity distribution in controls and PD is very similar with a peak at 10 min and a progressive decrease until the end of the acquisition. The TACs corresponding to the occipital cortex and cerebellum are overlapping for control and patient.

Regarding the cerebral kinetics results obtained in the controls, with stability of the [^18^F]LBT-999 striatal uptake from 20 min post-injection to the end of the study, and the evidence of an acceptable *in vivo* metabolism, ratios between the striatum and the cerebellum were calculated using a 10-min image acquired between 30 and 40 min post-injection (20).

On Table 1, mean ± SD for BP_ND_, DVR, and ratio values obtained for caudate and putamen nuclei and SN in controls and PD patients are shown. In PD patients, for striatal structures, BP_ND_, DVR, and ratios were significantly decreased compared with the control group (*p* < 0.001). This decrease is, as expected, more pronounced for the putamen (*p* < 0.001 and 0.01 for putamen and caudate, respectively) and especially for the most affected putamen. This decrease is obviously asymmetrical taking into account the more affected striatal side. However, for SN no significant asymmetry between the two sides is evidenced. No significant differences between BP_ND_ and DVR-1 are found (*p* > 0.5).

**Table 1:** BP_ND_ and DVR (using the dynamic 89 min acquisition) and ratios (calculated from the image acquired between 30 and 40 min post-injection).

		**Dynamic acquisition**		**30–40 min**
		**BP_**ND**_**	**DVR-1**	**Ratio**
HC	Caudate	6.3 ± 1.2	5.2 ± 0.9	5.0 ± 0.7
	Putamen	6.8 ± 1.2	5.6 ± 1.0	5.4 ± 0.7
	SN	1.3 ± 0.5	1.2 ± 0.5	2.1 ± 0.5
PD	Caudate more affected	3.5 ± 1.5	3.0 ± 0.8	3.5 ± 0.8
	Caudate less affected	4.1 ± 1.4	3.8 ± 1.0	4.0 ± 0.9
	Putamen more affected	2.1 ± 1.4	1.9 ± 0.9	2.8 ± 0.7
	Putamen less affected	3.0 ± 1.8	2.8 ± 1.2	3.4 ± 0.9
	SN more affected	0.6 ± 0.3	0.5 ± 0.3	1.5 ± 0.3
	SN less affected	0.7 ± 0.3	0.6 ± 0.2	1.5 ± 0.3

*For patients with Parkinson's disease, we present the values for the more and the less decreased uptake sides*.

*More affected and less affected sides correspond to the hemispheres contralateral and ipsilateral, respectively, to the less and more clinically affected hemibodies*.

*Values are mean ± SD*.

## Discussion

The results of the present study are not surprising and show, as expected, a decrease of the [^18^F]LBT-999 striatal uptake in parkinsonian patients. An asymmetrical striatal uptake according to the most affected clinical side was also observed. This is, as expected, more pronounced for the putamen. Concerning SN, we observed a quite symmetric uptake decrease. We should note that the partial volume effect has not been corrected, probably influencing the values calculated for SN

The activity of the occipital cortex is more often used as a non-specific reference region. In this study, we used the cerebellum based on the similarity of the cerebellar and occipital distribution of [^18^F]LBT-999 evidenced by the TACs. This suggests that these two regions may reflect the non-specific binding of the radioligand. Using three methods of estimation of DAT density, one of which is a simplified method using ratios and a 10-min image, a significant loss of dopaminergic pre-synaptic neurons PD was confirmed. Even using the ratios, and despite an underestimation of the neuronal loss, it is possible to distinguish patients with dopamine striatal damage from normal subjects.

One of the features that could influence the accuracy of the use of ratios is the time peak equilibrium. Unfortunately, for healthy controls, the peak of specific uptake reached at about 80–90 min post-injection, the duration of our acquisition (20). A longer acquisition could probably suggest a more appropriate PET acquisition time window. In any case, very long acquisitions (e.g., 3 h, even with a break) are difficult to accept by PD patients.

However, on the other hand, the metabolic results indicated that the unmetabolized plasmatic fraction of [^18^F]LBT-999 is predominant up to 45 min post-injection, leading us to propose as a compromise a simple image of 10 min acquired between 30 and 40 min post-injection, certainly well tolerated by the majority of the patients (20).

Another major limitation is that we were unable to take arterial samples that would allow us to validate the quantification methods used. In the validation of any new radioligand, this step is undoubtedly fundamental. We have assumed that either the cerebellum or the occipital cortex can be used as non-specific reference based on studies with other DAT radioligands. We also did not perform tests/retest PET, which could have contributed to the validation of the methods used.

Another important limitation of this study is that we did not compare [^18^F]LBT-999 with other DAT PET radioligands in the same subjects. In fact, other fluorine-18-labeled DAT radioligands have been developed. Among them are [^18^F]FP-CIT and [^18^F]FECNT. However, although the former has a concomitant affinity for dopamine and serotonin transporters, the latter presents radiolabeled metabolites that cross the blood–brain barrier with cerebellar accumulation (22, 23).

In the desire to get a tracer targeting the dopamine transporter, our laboratory was involved in the design and the synthesis of new molecules. Among the first compounds developed, PE2I has a very high affinity for the DAT associated with a very good selectivity regarding other monoaminergic transporters (24). This molecule, either radiolabeled with iodine-123 or carbon-11, has been used in many studies in both animals and humans (25, 26). The main limitation of PE2I in PET imaging is that it can only be used by hospitals equipped with a cyclotron owing to the half-life of carbon-11.

In the perspective of having a tracer that can be used more broadly, we invested in the development of analogous of PE2I radiolabeled with fluorine-18. Two molecules have been prepared: LBT-999, the radioligand used in this work, and FE-PE2I. These two tracers, both cocaine derivatives with similar DAT affinity, share the aforementioned structural elements that are decisive to be very potent and selective DAT tracers (9 nM for LBT-999 and 12 nM for FE-PE2I) (18, 27). However, the strategy for obtaining the two radioligands is quite different leading to production times of about 90 min for the [^18^F]FE-PE2I (28) compared with 30 min for [^18^F]LBT-999 which is more compatible with a production on an industrial scale (17).

Because FE-PE2I and LBT-999 share similar chemical structures, their *in vitro* and *in vivo* metabolisms are similar (29–31). However, compared with [^18^F]FE-PE2I, whose metabolism produces five radiometabolites, [^18^F]LBT-999 produces only three radiometabolites that can potentially interfere with image quality. However, the large number of studies in humans using [^18^F]FE-PE2I show that, despite this metabolism, fast DAT imaging is possible without observable interference as observed in the present work using [^18^F]LBT-999 (32).

We also did not compare [^18^F]LBT-999 to the reference radioligand used in clinical routine in the majority of nuclear medicine centers. Nevertheless, because it is an iodine-123-labeled tracer ([^123^I]FP-CIT), we must consider the intrinsic characteristics of both nuclear imaging systems. The intrinsic performance of PET compared with SPECT, in terms of spatial resolution and sensitivity, led the exploration of extrapyramidal syndromes with a DAT PET radioligand to become more comfortable for patients: a significantly shorter time of study, with an interval of about 30 min between injection and PET acquisition, and an image acquisition during only 10 min. In fact, for many patients with extrapyramidal syndrome, an acquisition of 35–40 min (as it is necessary in SPECT using the only radiopharmaceutical with the mandatory permissions to clinical use) is often hard to be tolerated. Finally, using non-iodine-labeled DAT ligands, pre-medication to prevent thyroid uptake is not necessary.

Even an important number of limitations, the results of this proof-of-concept study show that [^18^F]LBT-999 could be used as an alternative to other DAT radiotracers to evaluate the integrity of pre-synaptic striatal dopaminergic fibers with a short image acquired only during 10 min.

Studies including a greater number of patients, with other pathologies with dopaminergic system impairment and if possibly comparing this radioligand with other DAT radioligands are still necessary before validation of [^18^F]LBT-999 for clinical routine use.

## Data Availability Statement

The database of this study has been saved by the clinical research department of the CHU of Tours, which cannot be modified but remains available if requested.

## Ethics Statement

The studies involving human participants were reviewed and approved by Regional Ethic Committee and the French Agency for Safety and Security for Medical Devices. The patients/participants provided their written informed consent to participate in this study.

## Author Contributions

M-JR, DG, and J-BD contributed for conception and design of the study. JV, SM, and NA contributed for radiopharmaceutical production and controls. M-JR, LB, J-PC, VG, and KM were responsible for acquisition of data. M-JR, J-PC, CT, and PE performed analysis of imaging data. M-JR, JV, KM, CT, PE, and DG wrote sections of the manuscript. M-JR wrote first draft and final version of the manuscript. M-JR, JV, DG, and PE were responsible for critical revision of the manuscript for important intellectual content. J-BD and DG obtained funding. M-JR and DG were responsible for study supervision. All authors contributed to the article and approved the submitted version.

## Conflict of Interest

J-BD works for Zionexa (formerly Laboratoires Cyclopharma). The remaining authors declare that the research was conducted in the absence of any commercial or financial relationships that could be construed as a potential conflict of interest.
